# A Novel Immune Evasion Strategy of *Candida albicans*: Proteolytic Cleavage of a Salivary Antimicrobial Peptide

**DOI:** 10.1371/journal.pone.0005039

**Published:** 2009-04-07

**Authors:** Timothy F. Meiller, Bernhard Hube, Lydia Schild, Mark E. Shirtliff, Mark A. Scheper, Robert Winkler, Amy Ton, Mary Ann Jabra-Rizk

**Affiliations:** 1 Department of Oncology and Diagnostic Sciences, University of Maryland – Baltimore, Baltimore, Maryland, United States of America; 2 Marlene and Stewart Greenbaum Cancer Center, University of Maryland Medical System, Baltimore, Maryland, United States of America; 3 Department of Microbial Pathogenicity Mechanisms, Leibniz Institute for Natural Product Research and Infection Biology, Hans-Knoell-Institute (HKI), Jena, Germany; 4 Friedrich Schiller University, Jena, Germany; 5 Department of Microbial Pathogenesis, Dental School, University of Maryland – Baltimore, Baltimore, Maryland, United States of America; 6 Department of Microbiology and Immunology, School of Medicine, University of Maryland – Baltimore, Baltimore, Maryland, United States of America; 7 Department of Biomolecular Chemistry, Leibniz Institute for Natural Product Research and Infection Biology, Hans-Knoell-Institute (HKI), Jena, Germany; 8 Department of Pathology, School of Medicine, University of Maryland – Baltimore, Baltimore, Maryland, United States of America; University College Dublin, Ireland

## Abstract

Oropharyngeal candidiasis is an opportunistic infection considered to be a harbinger of AIDS. The etiologic agent *Candida albicans* is a fungal species commonly colonizing human mucosal surfaces. However, under conditions of immune dysfunction, colonizing *C. albicans* can become an opportunistic pathogen causing superficial or even life-threatening infections. The reasons behind this transition, however, are not clear. In the oral cavity, salivary antimicrobial peptides are considered to be an important part of the host innate defense system in the prevention of microbial colonization. Histatin-5 specifically has exhibited potent activity against *C. albicans*. Our previous studies have shown histatin-5 levels to be significantly reduced in the saliva of HIV+ individuals, indicating an important role for histatin-5 in keeping *C. albicans* in its commensal stage. The versatility in the pathogenic potential of *C. albicans* is the result of its ability to adapt through the regulation of virulence determinants, most notably of which are proteolytic enzymes (Saps), involved in tissue degradation. In this study, we show that *C. albicans* cells efficiently and rapidly degrade histatin-5, resulting in loss of its anti-candidal potency. In addition, we demonstrate that this cellular activity is due to proteolysis by a member of the secreted aspartic proteases (Sap) family involved in *C. albicans* pathogenesis. Specifically, the proteolysis was attributed to Sap9, in turn identifying histatin-5 as the first host-specific substrate for that isoenzyme. These findings demonstrate for the first time the ability of a specific *C. albicans* enzyme to degrade and deactivate a host antimicrobial peptide involved in the protection of the oral mucosa against *C. albicans*, thereby providing new insights into the factors directing the transition of *C. albicans* from commensal to pathogen, with important clinical implications for alternative therapy. This report characterizes the first defined mechanism behind the enhanced susceptibility of HIV+ individuals to oral candidiasis since the emergence of HIV.

## Introduction

Immunocompromised individuals and particularly the HIV-infected constitute a population that is highly susceptible to an array of opportunistic infections. Among the opportunistic pathogens, the fungal species *Candida albicans* is perhaps the most successful in humans [1]. A commensal fungus frequently colonizing human mucosal surfaces, *C. albicans* has long been adapted to the human host and has evolved because of the specific demands of the human host environment. Distinctively, under conditions of immune dysfunction, colonizing *C. albicans* strains can become opportunistic pathogens causing recurrent mucosal infections and life-threatening disseminated infections with high mortality rates [2], [3].

The association between oral candidiasis and an immunocompromised state dates back to the 19^th^ century when oral candidiasis was recognized as “…… *always the consequence of a pre-existing morbid state*.” This association became quite evident with the emergence of AIDS in the early 1980s. In HIV+ individuals, oropharyngeal candidiasis is considered an AIDS-defining illness with 80–90% of these individuals suffering recurrent episodes during the course of their illness [2], [4], [5]. The increasing emergence of strains of *C. albicans* resistant to the commonly used antifungal agents has made clinical management of candidiasis increasingly difficult and the need for improved drug therapies crucial [2], [6].

The oral cavity is a unique environment and a primary target for opportunistic infections, particularly candidiasis [4], [6]. The oral mucosa constitutes a critical protective interface between external and internal environments that serves as a barrier to the hundreds of microbial species present in this environment [4], [6]. In the oral cavity, saliva, a complex mix of fluids from salivary glands plays an important role in the maintenance of oral mucosal health [7], [8]. Specifically, saliva contains a set of antimicrobial peptides produced by the host which are considered to be an important part of the innate immune system, contributing to maintaining the balance between health and disease in this complex environment [9], [10]. Surprisingly, the important role of these natural antimicrobials in the protection of the oral cavity from the constant exposure to microbial challenges and particularly their potential as therapeutic agents, is only just beginning to be appreciated.

Most notable among the natural immune salivary antimicrobial peptides are the histatins, a family of low-molecular-weight, histidine-rich, cationic proteins produced and secreted by human parotid and submandibular-sublingual glands [11]–[13]. Histatins show killing activities against numerous oral bacteria, as well as potent antifungal properties against pathogenic fungi including *C. albicans* [12], [14], [15]. Histatin-5 (Hst-5) specifically, a 24-amino acid member of the family, has the highest level of activity against *C. albicans* including strains resistant to antifungal agents, implicating a different mode of action than the commonly used drugs [16], [17]. Histatin-5 is believed to exert its anti-candidal effect through binding to receptor proteins (Ssa1 and Ssa2) on the fungal cell membrane [18]. Once internalized, Hst-5 inhibits mitochondrial respiration, thus inducing the formation of reactive oxygen species thereby leading to mitochondrial and cytoplasmic membrane damage, efflux of ATP and cell death [10], [17], [19]. Recently, expansive investigations by Mochon *et al.* [20] provided direct evidence for a breach in plasma membrane as the initial damage by extracellular Hst-5 on *C. albicans* and a mechanism of its internalization into the cytoplasm.

Although not since substantiated, studies in the early 1990s had reported changes in salivary histatin concentrations in HIV+ individuals, the result of salivary gland dysfunction [5], [21]. Given the important role of saliva in maintaining oral health, it is conceivable that alterations in salivary gland secretion and/or composition are liable to contribute to the markedly enhanced predisposition of this population to oral candidiasis. Yet studies confirming these important observations have been lacking, most likely due to the lack of feasible methods for measuring salivary histatin concentrations. Recently, however, we confirmed these observations in a study comparing the levels of salivary Hst-5 between a group of HIV+ and HIV− individuals. Results from the investigation demonstrated significantly lower Hst-5 levels in the HIV+ group, concomitant with increased prevalence of *C. albicans* in the oral cavity, highlighting the involvement of host innate immunity in the protection against *C. albicans* colonization [22].

It is well-established that the process of development and course of microbial infections is regarded as an encounter between the virulence of a microorganism and the ability of the host to prevent microbial colonization or invasion. In the case of *C. albicans*, the transition from harmless commensal to disease-causing pathogen is finely balanced and attributable to an extensive array of virulence factors selectively expressed under suitable predisposing host conditions. This is quite evident through the diverse manifestations of candidiasis, for in addition to oral infections, *C. albicans* is currently ranked the third most commonly isolated bloodstream pathogen in hospitalized patients with a mortality rate of 40–50% [2], [23].

Most notable among the repertoire of virulence determinants expressed by *C. albicans* are a family of 10 proteolytic enzymes known as the secreted aspartic proteases (Saps) [24], [25]. The *SAP* gene family has been shown to be differentially expressed under a variety of laboratory growth conditions and during experimental *C. albicans* infections *in vitro* and *in vivo* [26]–[29]. The contribution of the Saps to mucosal and systemic infections and their involvement in adherence, tissue damage and evasion of host immune responses was clearly demonstrated using *SAP*-deficient mutants and protease inhibitors [25]. *Candida albicans* Saps have been shown to degrade a variety of host defense proteins such as lactoferrin and immunoglobulins [30]. A recent study by Villar *et al.* [31] demonstrated that *C. albicans* is able to degrade E-cadherin, the major protein in epithelial cell junction and that the degradation was mediated by Sap5. Therefore, the Sap isoenzymes appear to have a variety of functions *in vivo* which are probably called upon at different stages and in different types of *C. albicans* infections [25], [32].

Although the majority of the Sap enzymes are secreted by the fungus, the most recently identified members, Sap9 and Sap10 are cell surface glycosylphosphatidylinositol (GPI)-anchored proteases located in the fungal cell membrane and cell wall that have been described to be similar to the *S. cerevisiae* yaspin Yps1[32], [33]. In contrast to the secreted Saps (Saps1–8), no known specific host substrate has been identified for these proteases and consequently they were considered to target only proteins of fungal origin [32]. Precisely, because of their location and due to the fact that mutants lacking these proteases were hypersensitive to cell surface disturbing agents and similar to the *S. cerevisiase* Ysp1, the Sap9 and Sap10 proteases were linked to regulatory processes on the fungal cell surface with functions necessary for cell surface integrity [32], [33].

Despite the extensive available information on the association of *C. albicans* Saps and host protein degradation, in depth investigating into the ability of the Saps to degrade small salivary antimicrobial peptides specifically those with potent anti-candidal properties such as histatins, has not been fully investigated. A study by Ruissen *et al.* [34] had reported the susceptibility of Hst-5 to proteolysis by *C. albicans*. Although the secreted *C. albicans* enzymes were not found to be the responsible for the observed proteolysis in that study, a specific *C. albicans*-substance was not identified.

The identification of histatins as substrates to the Sap enzymes and the identification of a specific *C. albicans*-factor the maybe involved in evasion of host immune defenses would be of great significance as it would implicate direct post-secretional proteolysis of histatin in its decreased salivary levels. Such findings would carry significant implications as they may contribute to our understanding of the quandary of the enhanced propensity of the HIV+ population to oral candidiasis. To that end, this study aimed to analyze the ability of *C. albicans* to degrade Hst-5 *in vitro* and to attribute the proteolytic activity to the secreted aspartic proteases.

## Results

### Degradation of Hst-5 by *C. albicans*

**Figure 1:**
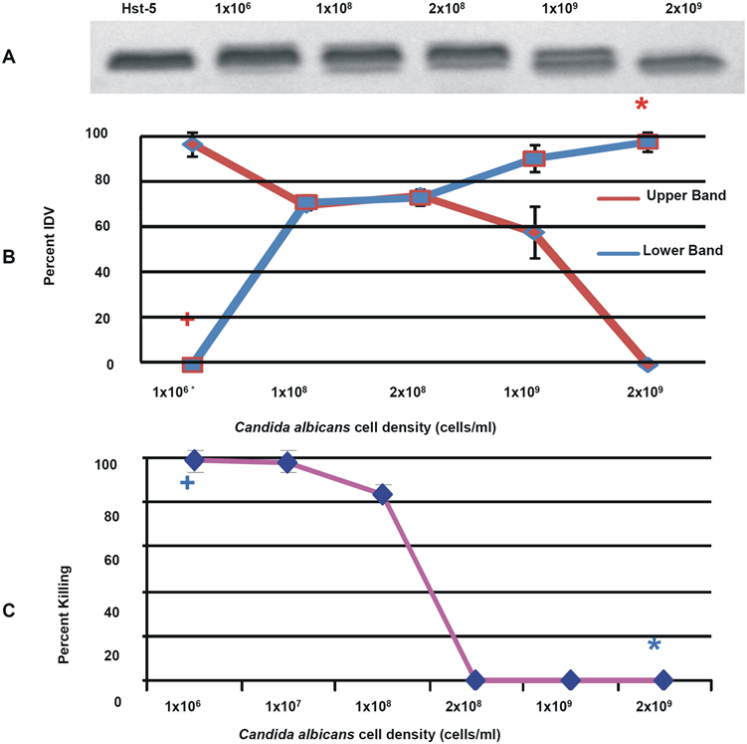
Degradation and killing potency of Hst-5. (A) Degradation of Hst-5 by *C. albicans* demonstrating degradation level proportional to cell density (cells/ml). (B) Decrease in the percent Integrated Density Value (IDV) of the intact Hst-5 (upper band) (P<0.02) and increase in density of degradation product (P<0.0001) (lower band). (C) Percent killing demonstrating significant killing + of *C. albicans* at 1×10^6^ cells/ml density correlating with no degradation + at that cell density; whereas no killing* at 2×10^9^ cells/ml correlates with significant degradation*. Error bars indicate the standard errors of the means.

**Figure 2:**
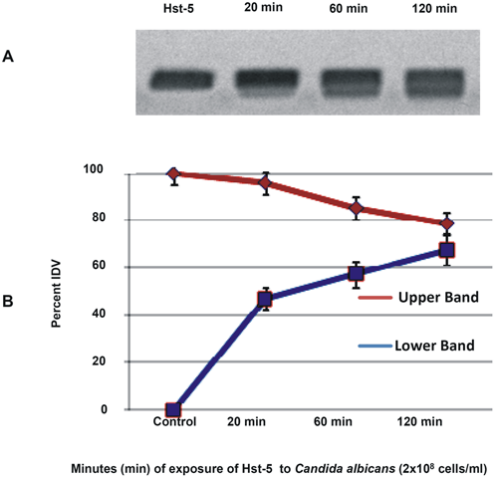
Degradation of Hst-5 by *C. albicans* over time (minutes) demonstrating degradation level proportional to exposure time. (A) Minor degradation following 20 mins incubation at 37°C observed by the appearance of lower weight product, whereas significantly increasing level of degradation seen following 1 h and 2 h incubation (B) Decrease in the percent of Integrated Density Value (IDV) of the intact Hst-5 (upper band) (P<0.0001) and increase in the IDV of the degradation product (lower band P<0.02) MW: molecular weight ladder.

**Figure 3:**
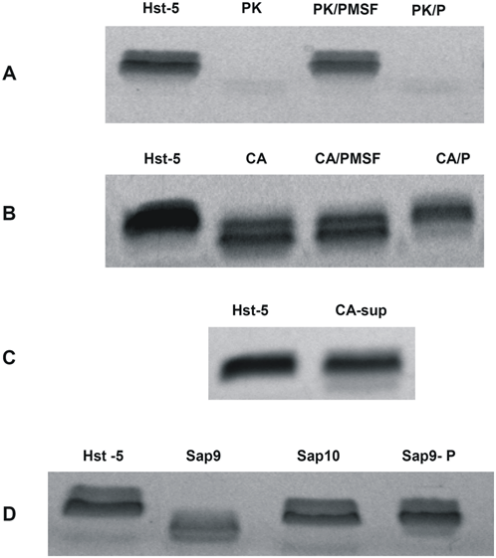
Degradation of Hst-5 under various conditions and *C. albicans* strains. (A) Complete degradation of Hst-5 by proteinase K (PK) used as positive control and inhibition of degradation by PMSF but not by pepstatin A (P) (B) Conversely, inhibition of *C. albicans* degradation by pepstatin A but not by PMSF (C) Minimal degradation observed when Hst-5 was incubated with *C. albicans* supernatant (CA-sup) D) Significant degradation of Hst-5 by 0.0625 µg Sap9 and Sap10 purified proteases and inhibition of degradation by pepstatin A.

*In vitro* degradation assays were designed to determine whether *C. albicans* possess the ability to degrade Hst-5. Following exposure of the peptide to *C. albicans* whole cells and cell-free supernatants of *C. albicans* cell suspensions, degradation reactions were subjected to electrophoretic analysis. Images revealed a gradual loss of peptide integrity proportional to the *C. albicans* cell density (Figure 1A and 1B) and time of exposure (Figure 2) demonstrating that *C. albicans* cells efficiently and rapidly degrade Hst-5. Furthermore, using a range of Hst-5 concentrations, the level of degradation was shown to be inversely proportional to Hst-5 concentration (50–200 µg/ml) (data not shown). Experiments to determine whether the proteolytic substance is secreted by *C. albicans* demonstrated significantly less degradation of Hst-5 by cell-free supernatants compared to whole *C. albicans* cells (Figure 3C).

### Inhibition of degradation by aspartic protease inhibitor

Proteinase K used as positive control for degradation completely degraded Hst-5 and degradation was inhibited by the serine protease inhibitor PMSF but not by the aspartic- specific inhibitor pepstatin A (Figure 3A). In contrast, PMSF failed to inhibit *C. albicans* degradation whereas pepstatin A resulted in inhibition of degradation indicating that the *C. albicans*-associated proteolytic substance is aspartic in nature (Figure 3B).

### Degradation of Hst-5 by *C. albicans* purified proteases

Purified proteases representing the various subfamilies of *C. albicans* Saps were tested for their ability to degrade Hst-5. Results demonstrated that Sap9 resulted in significant proteolysis of Hst-5 and to a lesser degree Sap10 protease, under all conditions tested and that the degradation was inhibited by pepstatin A. (Figure 3D). Sap5 protease did not degrade Hst-5 (data not shown), however Sap2 protease extensively degraded Hst-5 (Figure S1).

### Degradation of Hst-5 by *C. albicans SAP* mutants

**Figure 4:**
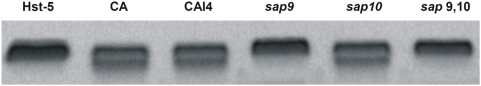
Minor inhibition of Hst-5 by the *SAP10* deficient null mutant (*sap10*) and significant inhibition by the *SAP9*-deficient mutant (*sap9*) and the *SAP9* and *SAP10* double mutant (*sap9,10*) compared to their parent strain (CAI4) and the *C. albicans* SC5314 (CA) strain.

To identify the Sap isoenzyme responsible for the degradation, *C. albicans SAP* null mutants were tested for their ability to degrade Hst-5. Results demonstrated no noticeable inhibition in degradation with the *sap1–3* and *sap4–6* mutants (data not shown), however, with the mutants lacking the cell surface-anchored proteases some degree of inhibition was seen with the *sap10* mutant, whereas complete loss of degradation was observed with the *sap9* single and the *sap9,10* double mutants (Figure 4) compared to their parent strain (CAI4) and the *C. albicans* SC5314 strain.

### Peptide sequencing of degradation products by MALDI-TOF/TOF MS analysis

**Figure 5:**
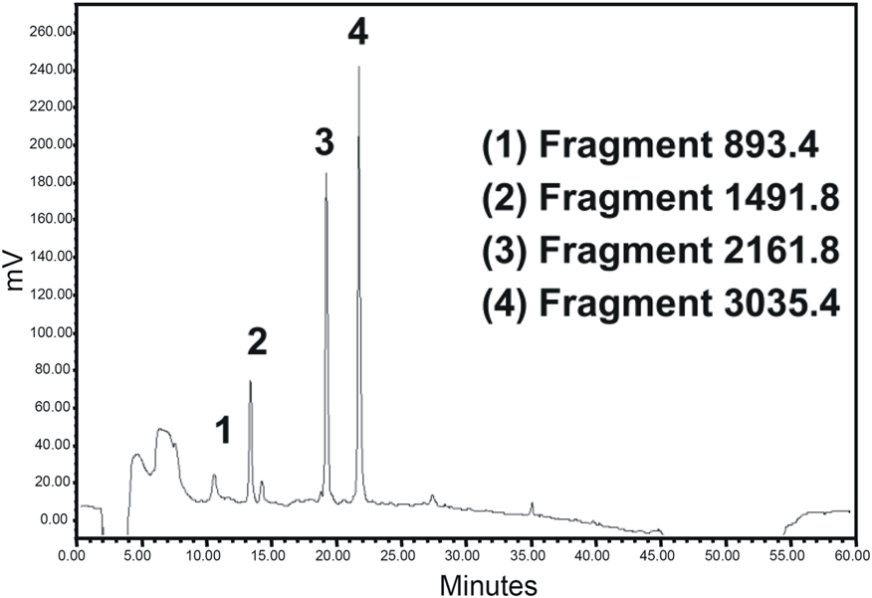
Separation of Hst-5 fragments by RP-HPLC following degradation by *C. albicans* resulting in 4 peaks corresponding to fragments 18–24, 1–12, 1–17, and 1–24 on the Hst-5 amino acid sequence.

**Figure 6:**
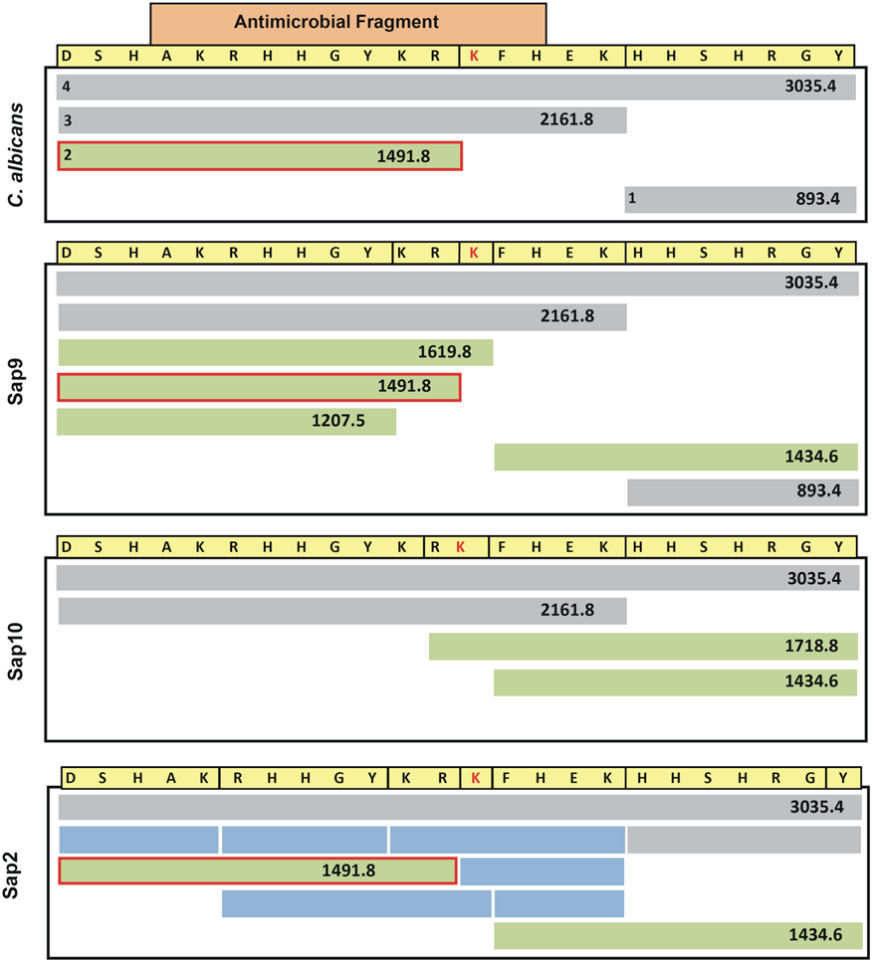
Comparison of cleavage fragments (1–4) identified by Mass Spectrometry following degradation of Hst-5 with *C. albicans* whole cells and purified Sap9, Sap10 or Sap2 proteases. The boxed fragment in orange represents the part of the peptide with antimicrobial properties. The green bars represent the fragments generated from cleavage within the antimicrobial fragment. The green bars boxed in red are the 1491.8 Da fragments generated by cleavage before Lys_13_ by *C. albicans* cells and Sap9. The blue bars represent the fragments generated only by Sap2 protease.

In order to determine the peptide sequences of the cleaved fragments, fragmentation products from the various degradation reactions with *C. albicans* cells and purified proteases were subjected to Mass Spectrometry. MALDI analysis revealed the presence of four peaks corresponding to 4 fragments generated following degradation of Hst-5 by *C. albicans* cells (Figure 5). The profile of the degradation by *C. albicans* most closely resembled that of the Sap9 protease (Figure 6). Specifically a 1491.8 Dalton (m+H^+^) fragment generated by *C. albicans* cleavage was present only in the reactions with the Sap9 protease. Interestingly, although the Sap5 protease did not degrade Hst-5 (data not shown), the Sap2 protease extensively degraded Hst-5 (Figure 6, Figure S1).

### Killing assay

In order to determine the anti-candidal potency of Hst-5, percent killing of *C. albicans* by Hst-5 was determined. The results from the killing assays demonstrated that the susceptibility of *C. albicans* to Hst-5 was inversely proportional to its cell density. At *C. albicans* density unable to significantly degrade Hst-5 (Figure 1A), >90% (p<0.05) of the cells were killed by Hst-5 (Figure 1C). Conversely, higher *C. albicans* cell densities shown to degrade Hst-5 (Figure 1A), were significantly less susceptible to killing by Hst-5 (Figure 1C).

More importantly, the location of cleavage sites for *C. albicans* and Sap9 within the antimicrobial fragment of Hst-5 as identified by peptide sequencing, implied that cleavage at these residues may affect the anti-candidal properties of Hst-5 (Figure 6). In order to validate the hypothesis, killing assays were performed to compare the potency of Hst-5 pre- and post-degradation by *C. albicans* cells and purified proteases.

Killing assays with the fragmented Hst-5 fragmented by *C. albicans* demonstrated 0% (p<0.05) killing ability compared to >90% prior to its cleavage. Similarly, degradation of Hst-5 by Sap9 and Sap10 purified proteases resulted in complete loss of Hst-5 killing ability.

Conversely, assays with Hst-5 following exposure to the *sap9,10* mutants did not affect its killing ability (p<0.05) corroborating the inability of these mutants to degrade Hst-5 thus preserving its killing potency as represented in Figure 3C.

Similarly, degradation reactions where pepstatin A was incorporated, not only salvaged Hst-5 from degradation by *C. albicans* but also maintained its ability to kill *C. albicans* (p<0.05). Combined, these findings directly associated the proteolysis of Hst-5 by *C. albicans* and purified proteases to loss of its killing activity and its preservation from degradation with conservation of its anti-candidal ability.

## Discussion

*Candida albicans* is a commensal fungus commonly colonizing human mucosal surfaces. However, under conditions of immune dysfunction, colonizing *C. albicans* can become an opportunistic pathogen causing recurrent infections [2], [4]. The versatility in the pathogenic potential of this fungal species is the result of its ability to adapt, evolve and evade host immune defenses through the regulation of virulence determinants, selectively expressed under suitable predisposing conditions [35]–[37]. These virulence factors may well vary, depending on the type of infection, the stage and site of infection and the nature of the host response. Most notable among the pathogenic features of *C. albicans* are the secreted aspartic proteases, a family of proteolytic enzymes considered to be vital for its pathogenesis [24], [25], [32].

The oral cavity is a prime target for infection by *C. albicans*. As a salivary peptide with potent anti-candidal properties, Hst-5 has been proposed to play an important role in protecting the oral mucosa from over-colonization by commensal *C. albicans* strains. Our previous investigations have demonstrated that HIV+ individuals, the population most vulnerable to oral candidiasis, have significantly decreased levels of Hst-5 in their saliva, concomitant with increased colonization with *C. albicans* [22]. These observations strongly supported the notion of an involvement for Hst-5 in the enhanced propensity of HIV+ individuals to this opportunistic infection [22].

Previous studies had reported the degradation of histidine-rich peptides by proteases. A study by Xu *et al.* [38] described loss of antifungal activity of histidine-rich peptides the result of their degradation by salivary protease. Similarly, Helmerhorst *et al.* [9] demonstrated that oral-fluid is capable of degrading Hst-5 *in vitro*. However, these studies attributed peptide degradation to intrinsic biological properties of saliva and the possibility of microbial proteases contributing to the observed degradation was not investigated. To that end, this study intended to demonstrate that *C. albicans* Saps are capable of degrading Hst-5 and to identify the specific isoenzyme(s) responsible for the proteolysis.

*In vitro* assays designed to assess the integrity of Hst-5 following exposure to *C. albicans* clearly demonstrated that *C. albicans* rapidly degrades Hst-5 in as little as 20 minutes (Figure 2A). In light of these findings and in order to attribute the degradation to the Saps specifically, we conducted experiments using: (1) cell-free supernatants of *C. albicans* suspensions in order to determine whether the proteolytic substance is secreted or cell-associated (2) specific protease inhibitors in order to determine the nature of the proteolytic substance (3) purified proteases to confirm the ability of the Saps to degrade Hst-5 *in vitro* and (4) *SAP*-deficient mutants in order to identify the isoenzyme(s) responsible for the degradation. The combined results from these experiments demonstrated that the *C. albicans*-produced proteolytic substance is cell-associated and aspartic in nature. The ability of the purified Sap proteases to efficiently degrade Hst-5, further implicated the Saps in the degradation of Hst-5. Interestingly, since the *C. albicans* Sap9 and Sap10 proteases are considered to be similar to the *S. cerevisiase* and *C. glabrata* yaspins, these species were tested for their ability to degrade Hst-5. Results from these experiments demonstrated that both *S. cerevisiase* and *C. glabrata* exhibited significant enhanced degradation of Hst-5 compared to *C. albicans* (Figure S2).

The availability of *SAP*-deficient mutants of *C. albicans* made it possible to identify the Sap family isoenzyme(s) responsible for the degradation of Hst-5. In these experiments, no visible differences in levels of degradation were observed with the *sap1–6* mutants indicating that these secreted isoenzymes are not responsible for the observed degradation under the conditions tested. In contrast, complete loss of degradation was observed with the *sap9* single mutant and the *sap 9,10* double mutant compared to the parent strain (Figure 4). These results directly implicated the Sap9 protease as the main isoenzyme responsible for the degradation of Hst-5 by *C. albicans* cells, corroborating the previous indications that the active proteolytic substance is cell-associated rather than secreted. Furthermore, a recent study demonstrated that among the *SAP* genes, *SAP9* is the most highly and consistently expressed gene during oral infections *in vivo* [28]. Combined these findings seem to ascribe an important role for Sap9 in oral candidiasis, as the increased expression of a proteolytic enzyme capable of efficiently degrading Hst-5 may be a key event in the transition from commensal to pathogenic growth or may further exacerbate the infectious process.

In order to identify the cleavage sites for *C. albicans* cells and purified proteases on Hst-5, peptide mapping was performed on fragmentation products following degradation of Hst-5. Matrix-assisted laser desorption/ionization mass spectrometry (MALDI-TOF/TOF MS) analysis demonstrated that the degradation profile generated by *C. albicans*, most closely resembled that of the Sap9 protease (Figure 6). More importantly, four of the identified cleavage sites were located within the 12-amino acid antimicrobial fragment of Hst-5, implying that cleavage at these sites would compromise the anti-candidal properties of Hst-5. These implications were confirmed following evaluation of the anti-candidal potency of Hst-5 following its degradation by *C. albicans* cell and Sap9 and Sap10 purified proteases. Compared to the 90% killing efficacy observed for the intact Hst-5, the fragmented Hst-5 had no effect on *C. albicans*. These findings clearly demonstrated that the ability of Hst-5 to kill *C. albicans* largely depends on its integrity.

In principle, the lack of the ability of the *sap9* mutant to degrade Hst-5 may imply that this mutant would be more susceptible to killing by Hst-5. Surprisingly, however, the mutant strain demonstrated no statistically significant differences in susceptibility to Hst-5 *in vitro*. The reasons are not clear, however, it is important to note that we have recently shown that the *sap9* and *sap10* mutants have altered cell surfaces and modified susceptibilities to antifungals [32]. Therefore, it is possible that the lack of enhanced susceptibility of the mutants to Hst-5 maybe the result of the cell surface modifications compensating for the lack of protease degradation by the mutants.

Adherence of *C. albicans* to oral tissue is a pre-requisite for colonization and proliferation leading to tissue invasion and infection [39], [40]. *Candida albicans* cell concentrations of 1×10^2^ cells/ml and below are indicative of commensal colonization in the oral cavity [39]. In our experiments, this cell density was shown to be susceptible and unable to degrade physiological concentrations of Hst-5 *in vitro*. However, our results demonstrated that the level of degradation of Hst-5 was proportional to the cell density of *C. albicans* implying that the role Hst-5 plays in the oral cavity is crucial in controlling the proliferation of commensal *C. albicans* strains and in turn, the over-colonization of the oral mucosa by the commensal strains.

It is interesting to note that the introduction of the HAART anti-HIV therapy considerably reduced the incidence of oral candidiasis [41]. This was not only attributed to the inhibition of HIV protease essential for virus processing and proliferation and therefore to an improved immunological status of a patient, but also to a direct inhibition of *C. albicans* proteases responsible for tissue invasion by the aspartic protease inhibitors part of the HAART cocktail. In light of our new findings, however, the observed protective effect of HAART may also be due to the direct inhibition of Sap9 specifically, thus protecting histatin from degradation and preserving its anti-candidal properties.

With the limited arsenal of antifungals available coupled with the increasing emergence of resistant strains, the prospect of preventing *C. albicans* colonization thus precluding candidiaisis by using innate peptide antimicrobials as alternative drug therapies is becoming increasingly attractive. Specifically, the anti-candidal property coupled with its lack of toxicity to human cells, makes Hst-5 a promising therapeutic agent for the treatment or prevention of oral candidiasis in immunocompromised individuals. However, in order to generate effective antimicrobial peptides, it is important to identify the requirements for their intracellular transport. In a recent study by Jang *et al.* [16], the substitution of two lysine residues (Lys_5_ and Lys_13_) in Hst-5, resulted in inhibition of Hst-5 intracellular transport into the fungal cell and in turn loss of killing function. These findings are significant as they demonstrate that translocation is dependent on the sequence of the imported peptide [16]. Interestingly, a study by Ruissen *et al.* [34] had reported that blocking cell entry of Hst-5 and several of its peptide fragments greatly impeded their degradation by *C. albicans* suggesting that proteolysis occurs mainly intracellularly and is not used as a protective mechanism against Hst-5 activity. In contrast, our study demonstrated that *C. albicans* Sap9 cleaves Hst-5 at Lys_13_, the residue required for the successful intracellular uptake of Hst-5. In light of these findings, and given the cell-surface location of the Sap9, it is more likely that proteolysis of Hst-5 by *C. albicans* occurs on the cell surface. Combined with the killing assay results demonstrating loss of anti-candidal properties of Hst-5 the result of its degradation by *C. albicans*, our findings indicate that the proteolysis of Hst-5 by *C. albicans* constitutes a self-defense strategy against host defenses in the oral cavity. The dissemination of these new findings therefore, is crucial for the design of novel peptides for therapeutic use for candidiasis and specifically those peptides based on the structure of histatins, which has been the focus of much research lately.

In summary, the novel findings from this investigation demonstrate the ability of *C. albicans* to degrade the salivary anti-candidal peptide Hst-5 in the process identifying Hst-5 as the first host-specific substrate to *C. albicans* Sap9. These findings suggest that Sap9 not only targets fungal-specific proteins, but also host targets, specifically those involved in innate immune defenses. Whereas the secreted members of the proteolytic enzymes are known to be responsible for tissue penetration, in the oral cavity the GPI-anchored proteases may pave the way to tissue invasion by targeting the host substrate comprising the first line of defense against invading pathogens. These findings warrant further in depth investigations in order to determine the exact mechanism of Hst-5 inactivation by *C. albicans*.

This report characterizes the first defined mechanism behind the enhanced susceptibility of HIV+ individuals to oral candidiasis since the emergence of HIV. The discussion and analysis presented provide new insights characterizing a dynamic process involving both host and pathogen factors behind the transition from commensalism to parasitism that ultimately leads to the development of oral candidiasis.

## Materials and Methods

### Reagents, strains, and growth conditions

The *C. albicans* SC5314 strain, the *C. albicans SAP* null mutants *sap1–3* triple mutant (lacking *SAP1–3*), *sap4–6* triple mutant (lacking *SAP4–6*), *sap9* single mutant (lacking *SAP9*), *sap10* single mutant (lacking *SAP10*) and *sap9,10* double mutant (lacking both *SAP9* and *SAP10*), as well as the parent strain CAI4 (*URA*-) were used in these experiments [42], [43], [44]. Strains were grown in YPD broth (Difco Laboratories, Detroit, MI) overnight at 30°C with shaking and cells were equilibrated in fresh media to an optical density of 1.0 at OD_600_. High purity Hst-5, proteinase K, PMSF and pepstatin A were purchased from Sigma (Sigma-Aldrich Chemical, St. Louis, MO). Histatin-5 was reconstituted in 1 mM phosphate-buffered saline (PBS) and used at a final concentration of 150 µg/ml (within the range of physiological salivary concentration) in all experiments unless otherwise stated. All experiments were performed on at least three separate occasions.

### Sample preparation and degradation assay

Degradation assays were performed by incubating Hst-5 with 100 µl of standardized cell concentrations of the *C. albicans* strains in PBS for 2 h at 37°C. Unless otherwise stated, *C. albicans* strains were used in experiments at a cell concentration of 2×10^8^ cells/ml (generating two visible bands). Protease K was used as a positive control and negative controls with peptide and no *C. albicans* were included. Following incubation, samples were collected, heated at 100°C to remove proteolytic activity and analyzed by sodium dodecyl sulfate polyacrylamide gel electrophoresis (SDS-PAGE). Experiments were also performed using increasing *C. albicans* cell densities and at different time points where samples were collected following 20, 60 and 120 minutes incubation. In addition, assays were performed using cell-free supernatants from *C. albicans* cell suspensions to determine whether the proteolytic substance is secreted.

### Densitometric analysis

The intensity of the bands on the Coomassie-stained gels was assessed by densitometric analysis using a MultiImager scanner (Bio-Rad Laboratories, Hercules California) and images analyzed with the PDQuest version 7.0 (BioRAd).

### Inhibition of degradation by protease inhibitors

Inhibition of degradation of Hst-5 by *C. albicans* cells was determined using the degradation assay as described above in the presence of 1 mM of the protease inhibitors pepstatin A and PMSF (Sigma).

### Degradation of Hst-5 by *C. albicans* purified proteases

The recombinant proteases Sap9 and Sap10 were produced in *Pichia pastoris* as described previously [32], [45]. Briefly, *P. pastoris* strain GS115 was transformed with the expression plasmid pKJ113 carrying truncated *SAP9* and *SAP10* versions lacking signal peptide- and GPI anchor consensus- encoding sequences. Proteases were recovered from culture supernatants and purified *via* anion exchange chromatography. Activity of proteases was verified by using a standard fluorescence casein based assay (Molecular Probes). Purified proteases (0.05, 0.1, 0.25 and 0.5 µg) were incubated with Hst-5 for 2 h. Following, samples were processed as described above.

### Peptide sequencing of degradation products by Matrix-assisted laser desorption/ionization mass spectrometry (MALDI-TOF MS)

Supernatants from the various degradation reactions were subjected to peptide mapping in order to determine the sequences of cleaved fragments, identify cleavage sites on the Hst-5 as well as other cleavage products that may not be visible by electrophoresis. Alternatively, fragments were first separated by RP-HPLC on a 1.0×150 mm Microsorb 300 C18 column prior to sequencing in order to reveal any other cleavage events that were refractory to direct sequence analysis. Peptide samples were mixed with saturated α-cyano-4-hydroxycinnamic acid in acetonitrile∶trifluoroacetic acid 0.1% (1∶2 *v/v*) then measured on an Ultraflex MALDI-ToF/ToF device using flexControl 3.0 for data collection and flexAnalysis 3.0 for spectra analysis and peaklist generation (Bruker Daltonics GmbH, Germany). Suitable peptides of the mass spectra were chosen for post source decay (PSD) MS analyses. For data interpretation the MS peaklists were imported into Biotools 3.0 (Bruker Daltonics) and compared to theoretical fragments from *in silico* digests of Hst-5, assuming different fragmentation mechanisms and taking into account missed cleavages.

### Histatin-5 killing assay

*Candida albicans* cells at various cell densities were mixed with Hst-5 in the wells of a 96-well microtiter plate and incubated for 2 h at 37°C with shaking. Negative controls with cells and PBS alone were included. Aliquots from reactions were inoculated on YPD agar and incubated for 24–48 h at 35°C. The number of single colonies on each plate was counted and percent cell killing calculated.

The antimicrobial efficacy of Hst-5 following its degradation was assessed where fragments liberated from degradation reactions were used in killing assays as described above. Percent killing was compared to that of intact Hst-5.

The ability of pepstatin A to maintain anti-candidal efficacy of Hst-5 through inhibition of its degradation was assessed by incorporating pepstatin A in the killing assays.

Killing assays were also performed following Hst-5 exposure to Sap purified proteases and *sap* mutants.

## Supporting Information

Figure S1 Degradation of Hst-5 by the Sap2 purified protease (A) gel image demonstrating the ability of Sap2 protease to degrade Hst-5 in a dose-dependent manner using 0.5 and 0.25 µg (B) cleavage fragments identified by MS analysis.
(0.33 MB TIF)

Figure S2 Degradation of Hst-5 by *Candida glabrata* (Cg) and *Saccharomyces cerevisiae* (Sc) in comparison to *C. albicans* (CA). Both species produce multiple (GPI)-linked aspartyl proteases, orthologues of the *C. albicans* Sap9 and Sap10 and, as shown, exhibit significant enhanced degradation of Hst-5 compared to *C. albicans*. Interestingly, *Candida glabrata* and *Saccharomyces cerevisiae* also exhibited enhanced resistance to Hst-5. These findings corroborate those implicating the GPI-anchored proteases in *C. albicans* in the degradation of Hst-5.
(0.27 MB TIF)
