# Supplementary figures and images for: A Novel Immune Evasion Strategy of Candida albicans: Proteolytic Cleavage of a Salivary Antimicrobial Peptide
Source: PLoS One. 2009 Apr 7;4(4):e5039. doi: 10.1371/journal.pone.0005039 (PMC2661360; doi:10.1371/journal.pone.0005039)

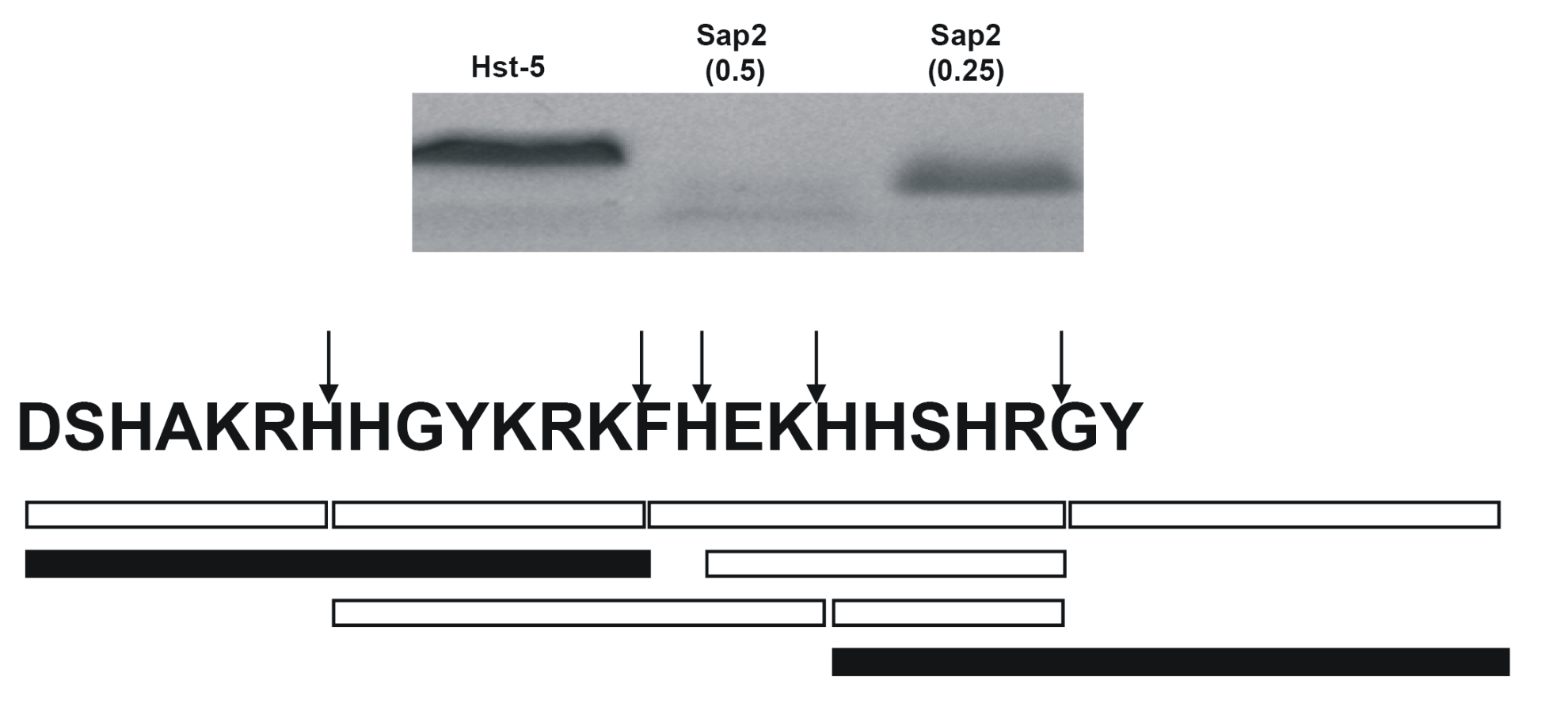

Supplement: Figure S1 — Degradation of Hst-5 by the Sap2 purified protease (A) gel image demonstrating the ability of Sap2 protease to degrade Hst-5 in a dose-dependent manner using 0.5 and 0.25 µg (B) cleavage fragments identified by MS analysis. (0.33 MB TIF) [file pone.0005039.s001.tif]

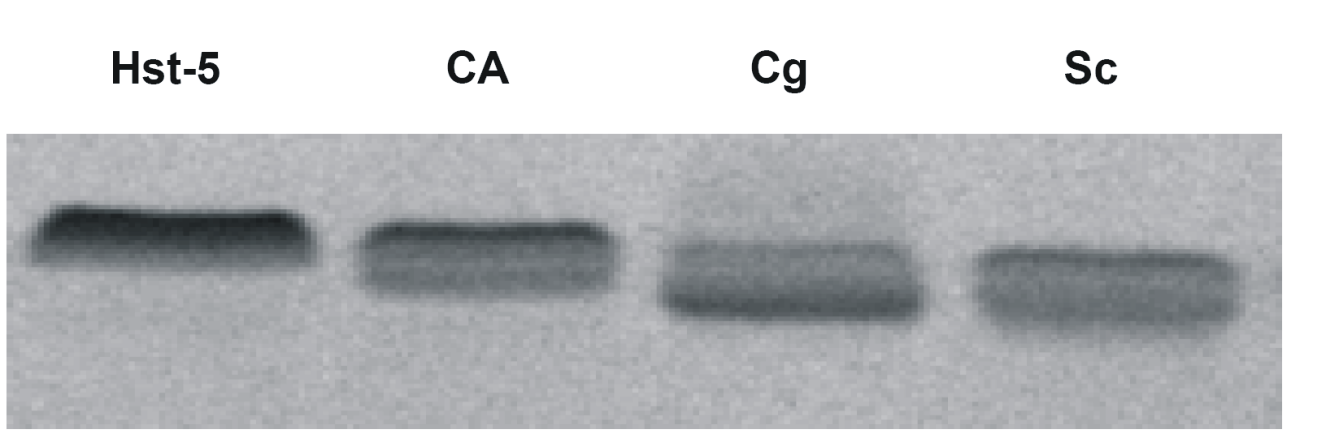

Supplement: Figure S2 — Degradation of Hst-5 by Candida glabrata (Cg) and Saccharomyces cerevisiae (Sc) in comparison to C. albicans (CA). Both species produce multiple (GPI)-linked aspartyl proteases, orthologues of the C. albicans Sap9 and Sap10 and, as shown, exhibit significant enhanced degradation of Hst-5 compared to C. albicans. Interestingly, Candida glabrata and Saccharomyces cerevisiae also exhibited enhanced resistance to Hst-5. These findings corroborate those implicating the GPI-anchored proteases in C. albicans in the degradation of Hst-5. (0.27 MB TIF) [file pone.0005039.s002.tif]
